# The progress and challenges of mesenchymal stromal cell-based therapy for diabetes and its complications

**DOI:** 10.1186/s40659-026-00687-w

**Published:** 2026-03-16

**Authors:** Leyi Tu, Tianyun Gao, Pingping Shen, Bin Wang

**Affiliations:** 1 Clinical Stem Cell Center, Nanjing Drum Tower Hospital, Affiliated Hospital of Medical School, Nanjing University, Nanjing, P. R. China; 2 State Key Laboratory of Pharmaceutical Biotechnology, Department of Urology, The Affiliated Nanjing Drum Tower Hospital, The Affiliated Hospital of Nanjing University Medical School, School of Life Sciences, Nanjing University, Nanjing, 210023 China

**Keywords:** Cell therapy, Diabetes, Mesenchymal stromal cells, Regenerative medicine, Translational research

## Abstract

**Background:**

Diabetes, a metabolic disorder characterized by chronic hyperglycemia resulting from insulin deficiency or resistance, continues to pose significant global health challenges with rising morbidity and mortality rates. While current therapies provide symptomatic management, they fail to address the underlying pathophysiology or prevent disease progression. Mesenchymal stromal cells (MSCs) have emerged as a promising therapeutic approach due to their unique triad of capabilities: multilineage differentiation potential, self-renewal capacity, and potent immunomodulatory properties. Through sophisticated paracrine mechanisms, MSCs exert multifaceted therapeutic effects including angiogenesis promotion, anti-inflammatory action, tissue regeneration, and immune system regulation, positioning them as ideal candidates for diabetes intervention.

**Scope of review:**

This comprehensive review provides three novel contributions to the field: (1) First systematic comparison of MSC tissue sources, including umbilical cord (UC-MSCs), bone marrow (BM-MSCs) and Wharton's jelly (WJ-MSCs), for diabetes treatment efficacy; (2) Critical analysis of recently developed MSC engineering techniques to enhance therapeutic outcomes; (3) Original synthesis of optimal delivery protocols for specific diabetic complications including diabetic wounds (DW), nephropathy (DNp), retinopathy (DR), neuropathy (DNe), and cardiomyopathy (DCM). Our analysis incorporates the most recent clinical trial data (2020–2023) not covered in previous reviews.

**Major conclusions:**

Combined with preclinical studies and clinical trials, MSC therapy demonstrates significant safety and efficacy. However, therapeutic durability remains limited, with efficacy declining by 40**–**60% after 6 months in most trials. This raises significant questions about its long-term clinical impact. This limitation stems primarily from poor cell engraftment and compromised survival and function within the hostile diabetic microenvironment, which is characterized by chronic inflammation, oxidative stress, and endoplasmic reticulum stress. Emerging solutions include: (1) MSC preconditioning with hypoxia; (2) Biomaterial encapsulation; and (3) Genetic modification for targeted delivery.

## Introduction

Diabetes is a chronic metabolic disease characterized by persistent hyperglycemia and has emerged as a leading cause of global morbidity and mortality [1]. According to the International Diabetes Federation, approximately 537 million adults worldwide had diabetes in 2021, with projections indicating a 46% increase to 783 million by the year 2045 [2]. Notably, low- and middle-income countries—particularly in Southeast Asia, South Asia, and the Middle East and North Africa—are experiencing the most rapid rise in prevalence, with Pakistani women demonstrating the most dramatic increase from 9.0% in 1990 to 30.9% in 2022 [3]. The disease exerts a profound burden on healthcare systems due to its association with life-threatening complications, including neuropathy, retinopathy, nephropathy, and cardiovascular diseases [4]. These complications arise from prolonged exposure to elevated blood glucose levels, contributing to increased disability and premature death.

Despite advances in diabetes management, current therapeutic strategies—including lifestyle modifications, oral hypoglycemic agents, and insulin therapy—remain insufficient in achieving long-term glycemic control without adverse effects [4].

Insulin, while life-saving for many patients, carries risks such as hypoglycemia, weight gain, and the potential for overtreatment [5]. Similarly, non-insulin pharmacological agents, such as metformin, sulfonylureas, thiazolidinediones, and sodium-glucose co-transporter 2 (SGLT 2) inhibitors, are limited by gastrointestinal disturbances, cardiovascular risks, and other side effects [6]. These challenges underscore the urgent need for novel therapeutic approaches that can restore metabolic homeostasis with minimal complications.

The unique immunomodulatory and regenerative profile of MSCs has prompted interest in their application for diabetes, holding promise for addressing key pathological processes [7]. The complex pathogenesis of diabetes involves not only insulin secretion defects and glucose metabolism disorders but also chronic inflammation, immune dysregulation, and micro/macrovascular complications. Current therapeutic regimens, including state-of-the-art insulin delivery technologies, provide only suboptimal glycemic control and fail to eliminate the risk of hypoglycemia, as they are fundamentally limited by their inability to restore functional β-cell mass or correct the underlying immune dysregulation. Although pancreatic islet transplantation demonstrates the conceptual feasibility of β-cell replacement, its broad application is constrained by donor scarcity, the frequent necessity for multiple transplants, and persistent challenges of immune rejection [8]. MSCs overcome these limitations via multi-target mechanisms: (1) Immune-metabolic regulation: MSCs restore immune homeostasis by secreting cytokines suppressing autoimmune-mediated β-cell destruction and alleviating chronic inflammation in insulin-target tissues [9, 10]; (2) Vascular and neural protection: Hyperglycemia-induced endothelial damage and neural ischemia drive diabetic complications. MSC-derived pro-angiogenic factors repair microcirculation, while exosomal nucleic acids (e.g., microRNA-126 (miR-126)) preserve endothelial function, delaying nephropathy, retinopathy, and neuropathy [11]; (3) β-cell regeneration: Preclinical studies demonstrate that MSC-exosomes deliver regenerative signals (e.g., Wnt4) to activate pancreatic progenitors while reducing oxidative stress-induced β-cell apoptosis, enabling functional islet mass recovery [12].

**Fig. 1:**
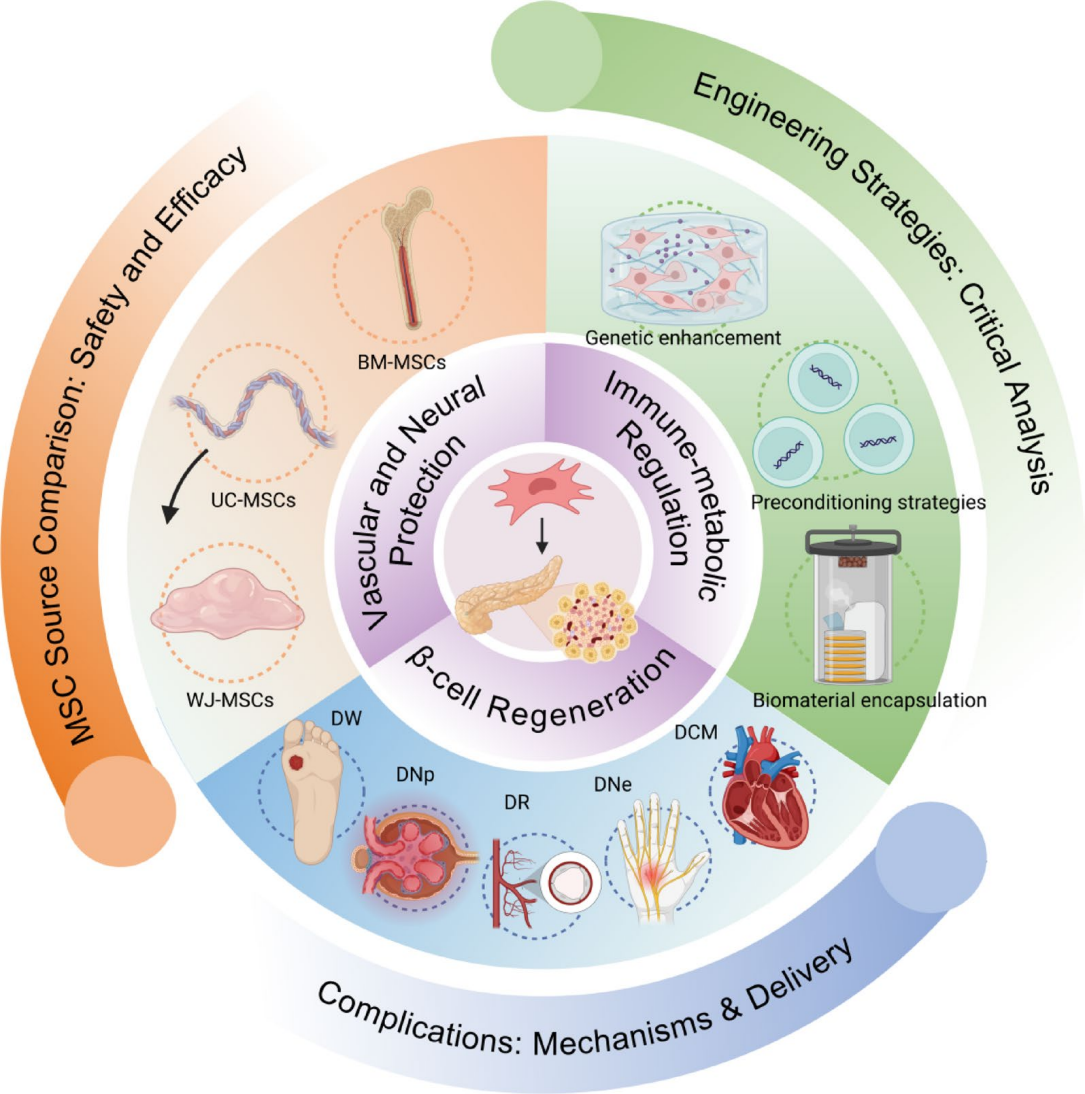
Overview diagram. Created in BioRender. Tu, L. (2025) https://BioRender.com/s7q9i2r

This multi-pronged therapeutic profile suggests MSCs could potentially address diabetes pathogenesis beyond symptomatic management. This review systematically examines the current evidence regarding MSCs in diabetes therapy, focusing on their mechanisms of action, preclinical findings, clinical trial outcomes, and persistent challenges. Our analysis of peer-reviewed literature from PubMed, Web of Science, and Scopus prioritizes randomized controlled trials and original research published within the last decade. By critically synthesizing this evidence, this review aims to evaluate the current state and future prospects of MSC-based therapies for diabetes treatment fig. 1.

## Overview of diabetes

### Pathophysiology of diabetes

**Fig. 2:**
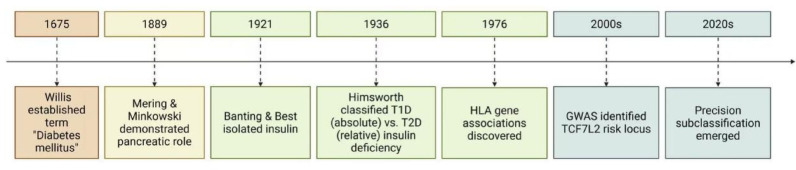
Pathological progression of diabetes. Created in BioRender. Tu, L. (2025) https://BioRender.com/m5a1q8e

The understanding of diabetes has evolved from ancient observations of sweet urine to modern molecular insights, as shown in Fig. 2. The pancreatic crucial role in glucose metabolism was established by Mering and Minkowski's 1889 pancreatectomy experiments [13, 14]. The landmark isolation of insulin by Banting and Best (1921) revealed insulin deficiency as the core defect in type 1 diabetes (T1D), while subsequent research identified insulin resistance and β-cell dysfunction as central to type 2 diabetes (T2D) pathogenesis [15, 16]. Modern research highlights three key pathophysiological aspects particularly relevant to MSC therapy: (1) β-cell destruction: In T1D, autoimmune-mediated β-cell loss leads to absolute insulin deficiency [17]; (2) Metabolic dysregulation: T2D involves peripheral insulin resistance, excessive hepatic glucose production, and progressive β-cell failure [18]; (3) Systemic dysfunction: Chronic inflammation, endoplasmic reticulum stress, and epigenetic modifications contribute to disease progression [19]. These pathophysiological insights inform the development of MSC-based therapies aimed at restoring β-cell mass, modulating immune responses, and improving insulin sensitivity—addressing the root causes rather than just symptoms of diabetes, as shown in Table 1.

**Table 1:** MSC-based treatment strategies for different diabetes types

Core MSC-based strategy	Type of diabetes	Primary pathological features
Immunomodulation & β-Cell Protection	T1D	(1) β-Cell Destruction Autoimmune-mediated apoptosis/loss of β-cells; Leads to absolute insulin deficiency
Metabolic Improvement & Function Preservation	T2D	(2) Metabolic Dysregulation Peripheral insulin resistance (muscle, adipose); Hepatic glucose overproduction; Progressive β-cell functional failure
Cell Replacement & Tissue Engineering	Late-Stage T1D & T2D	The Common Consequence of (1) & (2) Severe, irreversible loss of β-cell mass
Fundamental MSC Mechanisms	All Types of Diabetes	(3) Systemic Dysfunction Chronic inflammation; Endoplasmic reticulum stress; Epigenetic modifications

### Classification of diabetes

According to the "Classification and Diagnosis of Diabetes" issued by the American Diabetes Association, diabetes can be classified into four main categories: T1D, T2D, gestational (GDM), and other specific types [20]. T1D (5–10% of cases) is caused by autoimmune destruction of β-cells by CD4^+^ effector T cells, leading to absolute insulin deficiency [21]. Autoantibodies against glutamic acid decarboxylase 65, insulin, and tyrosine phosphatases serve as diagnostic markers [22]. While typically lean, obesity doesn't exclude T1D diagnosis [23]. T2D (90–95% of cases) predominantly affects overweight/obese individuals and involves complex interactions between genetic predisposition, insulin resistance, and progressive β-cell dysfunction [24]. This results in relative insulin deficiency and makes β-cell preservation a key therapeutic target. GDM develops during pregnancy, affecting 14% of pregnancies globally [25]. Though often resolving postpartum, it signals metabolic dysfunction with implications for both mother and offspring [4]. Other types include: (1) monogenic diabetes (0.5–5% of cases) caused by single-gene defects (e.g., GCK, HNF1A) affecting β-cell function; (2) pancreatogenic diabetes from exocrine pancreatic damage; (3) drug-induced diabetes, though mechanisms remain unclear [26–28]. Emerging classification systems using cluster analysis of clinical and metabolic parameters may better predict treatment response and complications [29]. This refined categorization is particularly relevant for developing targeted therapies like MSCs, as different diabetes subtypes likely require distinct regenerative approaches based on their underlying pathophysiology (Fig. 2).

### Diagnosis of diabetes

Current diagnostic criteria for diabetes remain based on the World Health Organization 1999 guidelines. Diagnosis requires either random plasma glucose ≥ 200 mg/dL (11.1 mmol/L) or fasting plasma glucose ≥ 126 mg/dL (7.0 mmol/L) with classic symptoms [30].

## Application of MSC-based therapy in diabetes

### Molecular mechanisms of MSCs in diabetes treatment

**Fig. 3:**
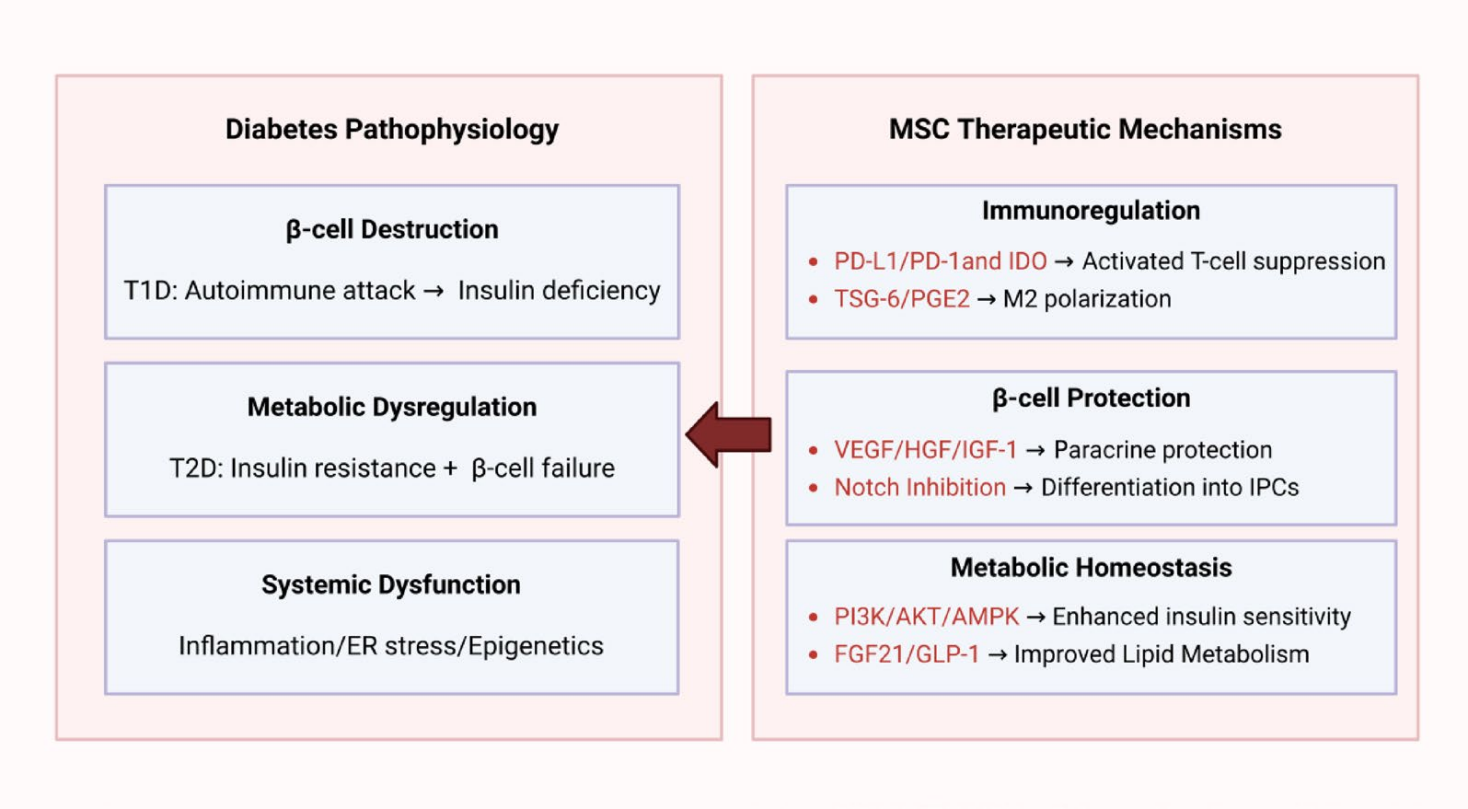
Molecular mechanisms of MSCs in diabetes treatment. Created in BioRender. Tu, L. (2025) https://BioRender.com/2x9byjp

MSCs exert therapeutic effects on diabetes through multimodal modulation of key pathological mechanisms, as shown in Fig. 3. Firstly, central to MSC therapy is their immunoregulatory capacity: MSCs regulate T cell homeostasis via the programmed death-ligand 1/programmed cell death protein 1 (PD-L1/PD-1) signaling axis and indoleamine 2,3-dioxygenase (IDO)-mediated metabolic reprogramming. Studies show that adipose-derived MSCs (AD-MSCs) significantly attenuated PD-1/PD-L1 blockade-induced diabetes incidence from 64 to 19% (*p* = 0.0065), by inhibiting the accumulation of autoreactive T cells and CXCL9 + macrophages in the islet β-cell regions [31]. Tumor necrosis factor-α (TNF-α) and interferon-γ preconditioning enhanced MSC function, upregulating IDO1 and prostaglandin E synthase 2 while secreting anti-inflammatory mediators including interleukin-6 (IL-6), IL-10 and prostaglandin E2 (PGE2). This suppressed activated T cell proliferation (CD4 + T cells: 21–5%; CD8 + T cells: 17–8%) and expanded regulatory T cell, mitigating autoimmune islet destruction [32]. By secreting PGE2 and tumor necrosis factor-inducible gene 6 protein (TSG-6), MSCs drive macrophage polarization to an anti-inflammatory M2 phenotype, which in turn establishes immune tolerance via IL-10/IL-4 release, PD-L1 expression, and C–C motif chemokine ligand 22 (CCL22)/CCL18-mediated recruitment of regulatory T cells [13].

Secondly, MSCs directly protect and regenerate β-cells through the paracrine pathway: they secrete vascular endothelial growth factor (VEGF), hepatocyte growth factor (HGF), and insulin-like growth factor-1, which exhibit anti-apoptotic and pro-regenerative functions. Deferoxamine-pretreated MSCs (DFX-CM) significantly upregulated VEGF secretion, markedly attenuating high glucose-induced β-cell apoptosis. In vivo, DFX-CM expanded pancreatic islet volume by 36% (*p* < 0.05) and increased insulin-positive area (64.2 ± 5.1% vs. 41.8 ± 4.3% in N-CM group; *p* < 0.05) [33].

Thirdly, under specific culture conditions, MSCs differentiate into functional insulin-producing cells (IPCs). Hu et al. first elucidated that Notch signaling inhibition enhanced IPC differentiation, increasing insulin gene expression by 3.1-fold (*p* < 0.001) and proinsulin protein expression by 2.6-fold (*p* < 0.02) [34]. However, the glucose-sensing capacity of IPCs remains suboptimal, highlighting the need for further protocol refinements.

Finally, MSCs restore metabolic homeostasis by promoting insulin sensitivity and regulating glucose metabolism in peripheral tissue. Nanovesicles derived from H8-BMSCs inhibited hepatic glucose-6-phosphatase and phosphoenolpyruvate carboxykinase expression via the PI3K/AKT/AMPK pathway, improving insulin resistance in T2D models [35]. In a complementary study, Xue et al. demonstrated that dual-gene (fibroblast growth factor 21/glucagon-like peptide-1 (FGF21/GLP-1)) engineered MSCs activated the invariant natural killer T cell-FGF21 axis and suppressed sterol regulatory element-binding protein 1/2 expression, resulting in significant amelioration of insulin resistance and glucose/lipid metabolic dysregulation [36].

In summary, MSCs provide a multi-target intervention strategy for diabetes through the trinity mechanism of "immune regulation**–**β-cell repair**–**metabolic remodeling," while engineering approaches such as DFX pretreatment can further optimize the efficacy of their secretome, promoting the transformation of cell therapy into a cell-free therapeutic paradigm.

### Clinical advances of MSC-based therapy for diabetes

#### Safety

A search of the ClinicalTrials.gov database using the strategy ("mesenchymal stromal cells" AND "diabetes") identified 111 registered clinical trials. These trials primarily utilized MSCs derived from umbilical cord, bone marrow, or Wharton's jelly sources. Information on the trials is collated in Table 2 [37–41] and Table 3 [42–47].

**Table 2:** MSC-based therapy for T1D in clinical trials

Diabetes	Subjects (n)	Stem cell type	Follow-up	Baseline characteristics	Key outcomes	Trial registration	References
T1D	42	UC-MSCs	1 year	Mean duration 8 years HbA1c ~ 8.6%	Significant metabolic improvements vs. control: 105.7% increase in AUCC-Pep (*p* = 0.00012) 49.3% elevation in insulin AUC (*p* = 0.01) 12.6% reduction in HbA1c (*p* < 0.01)	NCT01374854	[37]
	53	UC-MSCs	1 year	Disease duration 4.7 months Mean HbA1c 9.5%	40.7% of MSC-treated subjects met primary endpoint (vs. control, *p* < 0.05) 3 cases achieved insulin independence for 3–12 months (vs. 0 in control, *p* < 0.05)	ChiCTR2100045434	[38]
	42	UC-MSCs	8 years	Mean duration 8.1 years Mean HbA1c ~ 8.6%	Lower incidence of complications vs. control: DNe: 7.1% vs. 46.7% (*p* = 0.017) DNp: 7.1% vs. 40.0% (*p* = 0.039) DR: 7.1% vs. 33.3% (*p* = 0.081)	NCT01374854	[39]
	21	BM-MSCs	1 year	Newly diagnosed (within 6 weeks) C-peptide ≥ 0.3 nmol/L	Early MSC transplantation: Improved HbA1c and C-peptide levels Promoted an anti-inflammatory cytokine profile	NCT04078308	[40]
	24	WJ-MSCs	12 months	Mean duration 2 years Fasting C-peptide > 0.12 nmol/L	Attenuated C-peptide decline (10% vs. 47% in placebo, *p* < 0.05) Stabilized insulin needs (0 vs. + 10 U/day in placebo, *p* < 0.05)	NCT03406585	[41]

**Table 3:** MSC-based therapy for T2D in clinical trials

Diabetes	Subjects (n)	Stem cell type	Follow-up	Baseline characteristics	Key outcomes	Trial registration	References
T2D	91	UC-MSCs	48 weeks	Mean duration < 20 years HbA1c 7.0–12.0% Fasting C-peptide ≥ 1 ng/mL	vs. Placebo: Greater reduction in insulin dependence (27.78% vs. 15.62%, *p* < 0.05) Greater HbA1c reduction (1.31% vs. 0.63%, *p* = 0.0081) No significant improvement in pancreatic β-cell function	NCT02302599	[42]
	73	UC-MSCs	48 weeks	Mean duration ~ 11.6 years HbA1c ~ 8.8% Fasting C-peptide ~ 1.98 ng/mL	vs. Placebo: Higher TIR (26.54 vs. 15.84 by week 9; 21.36 vs. 6.32 by week 48) Greater HbA1c decreases (− 1.79% vs. − 0.96% by week 9; − 1.36% vs. − 0.51% by week 48)	NCT02302599	[43]
	34	UC-MSCs	24 weeks	Mean duration ~ 10 years HbA1c ~ 7.95%	vs. Placebo: Decreased lymphocyte counts and thrombin time (*p* < 0.05) Increased platelet counts, immunoglobulin levels, fibrinogen, D-dimer and neutrophil-to-lymphocyte ratio (all* p* < 0.05) No significant differences in tumor markers, lipid profiles or radiographic findings	ChiCTR2200057370	[44]
	73	UC-MSCs	48 weeks	Effective group vs. ineffective group: Duration 10 vs. 10.39 years HbA1c 9.16% vs. 8.38% Fasting C-peptide 1.84 vs. 2.19 ng/mL	Male patients with higher C-peptide AUC levels respond better to UC-MSC therapy	NCT02302599	[45]
	30	BM-MSCs	12 months	Mean duration 13–15 years HbA1c ~ 6.5–6.9% Fasting C-peptide 0.4–0.5 nmol/L	Both autologous BM-MSC and BM-MNC transplantation led to: ≥ 50% decrease in exogenous insulin dependence (*p* < 0.05) ~ 67% of patients maintaining HbA1c < 7.0% (53.0 mmol/mol, *p* < 0.05)	NCT01759823	[46]
	96	BM-MSCs	8 years	Mean duration ~ 8 years, HbA1c ~ 8.6–8.7% Fasting C-peptide 1.2–1.3 ng/mL	Year 1: C-peptide AUC increased significantly (Dual MSC + MNC: 50.6%; MNC-only: 32.8%) Year 8: Dual MSC + MNC group showed lower macrovascular complications (13.8%, *p* = 0.009) compared with MNC-only (21.4%,* p* = 0.061) and Control group (44.8%)	NCT01719640	[47]

Current evidence indicates that MSC therapy exhibits a favorable safety profile in the short term (median follow-up: 12–24 months). Adverse events (AEs) are primarily mild infusion-related reactions, most commonly self-limiting fever (incidence: 15–20%), which typically resolves spontaneously within 24–48 h post-infusion. No treatment-related serious adverse events, including theoretical risks such as tumorigenicity, ectopic tissue formation, or severe immune rejection, were observed during clinical trials [44]. Nevertheless, the long-term safety (> 5 years follow-up) warrants careful consideration. Potential concerns primarily involve unpredictable immune responses of allogeneic MSCs in specific microenvironments, the theoretical possibility of unintended differentiation of administered cells in vivo, and, in the case of extracellular vesicle-based therapies, the unknown long-term implications of their biodistribution and accumulation in organs such as the liver and spleen. Consequently, despite the established short-term safety, these uncertainties represent the key rationale for regulatory mandates requiring extended follow-up periods and further validation in larger-scale studies.

#### Effectiveness

Substantial clinical evidence has firmly established the multifaceted therapeutic efficacy of MSCs in diabetes management, with key outcomes consistently reaching clinically meaningful thresholds (e.g., HbA1c reduction ≥ 1%, insulin usage decrease ≥ 50%).

The efficacy in T1D has been particularly substantiated by multiple controlled trials. Among patients receiving UC-MSCs, 40.7% achieved ≥ 10% improvement in fasting C-peptide levels over 12-month follow-up (*p* < 0.05), including 3 patients who attained complete insulin independence for 3–12 months—an outcome unreported in control groups (NCT04078308) [40]. Most notably, the combination therapy of UC-MSCs with autologous bone marrow mononuclear cells (aBM-MNCs) delivered through pancreatic arterial infusion yielded striking metabolic benefits: a 105.7% increase in C-peptide area under the curve (AUCC-Pep; *p* = 0.00012), 49.3% higher insulin secretion (*p* = 0.01), and 12.6% reduction in glycosylated hemoglobin (HbA1c; *p* < 0.01) (NCT01374854) [37]. Eight-year longitudinal data further confirmed this regimen's capacity to significantly mitigate microvascular complications, including reduced incidence of DNe (7.1% vs. 46.7%; *p* = 0.017), DNp (7.1% vs. 40.0%; *p* = 0.039), and DR (7.1% vs. 33.3%; *p* = 0.081) (NCT01374854) [39]. Comparative analyses of administration routes indicated superior metabolic parameter improvement with pancreatic artery-targeted delivery, whereas single intravenous allogeneic UC-MSC infusion still induced clinical remission—supporting the feasibility of "off-the-shelf" cellular therapeutics.

While MSC-based therapy shows promising outcomes in T1D by addressing autoimmune-mediated β-cell destruction, its therapeutic potential extends to T2D—a condition characterized by insulin resistance and progressive β-cell dysfunction—where MSCs demonstrate distinct mechanisms of action and clinical benefits. Clinical trials indicate that intravenous infusion of UC-MSCs led to a notable reduction in HbA1c levels compared to placebo, with a maximum decrease of 1.79% observed at 9 weeks post-treatment, highlighting their early efficacy (NCT02302599) [43]. Continuous glucose monitoring further revealed that MSC-treated patients achieved higher time-in-range (TIR) values at both 9 weeks (26.54 vs. 15.84) and 48 weeks (21.36 vs. 6.32), underscoring improved glycemic stability [43].

Moreover, MSC therapy aids in the recovery of endogenous pancreatic β-cell function, with its effectiveness linked to patients' baseline islet status. Research suggests that male patients with elevated baseline C-peptide levels exhibit a more favorable response to UC-MSC treatment (NCT02302599) [45]. Additionally, combination therapies, such as BM-MSCs paired with BM-MNCs, demonstrate superior outcomes in islet function enhancement. After one year, the combination group showed a 50.6% improvement in AUCC-Pep, outperforming the MNC-only group (32.8%) (NCT01719640) [47].

In the field of diabetes treatment, MSCs have demonstrated significant therapeutic potential, yet their widespread application still faces numerous challenges. Firstly, the sample sizes of existing studies are generally small, and the follow-up periods are relatively short, making it difficult to comprehensively evaluate their long-term efficacy and safety. Secondly, the limited availability of tissue samples for validating protein expression levels somewhat restricts the depth and scientific rigor of the research. Thirdly, the majority of the included studies adopted an open-label design. Compared to double-blind randomized controlled trials, such a design is more susceptible to placebo effects and observer bias, especially when assessing subjectively strong "soft" endpoints, where such bias may lead to a significant overestimation of the treatment effect. Despite these limitations, MSCs have achieved preliminary success in improving glucose metabolism, protecting pancreatic β-cell function, and modulating immune responses, thereby opening new avenues for diabetes treatment. Moving forward, there is an urgent need for larger-scale, well-designed long-term follow-up studies to further clarify the therapeutic effects and safety profile of MSCs. This will provide a solid scientific foundation for their clinical translation and drive the development and maturation of this cutting-edge therapy.

#### Comparative analysis of advantages and limitations of MSCs derived from various sources

Current clinical research primarily utilizes UC-MSCs, BM-MSCs and WJ-MSCs, each with distinct therapeutic emphases. UC-MSCs excel in improving glucose metabolism metrics (e.g., HbA1c, TIR), while BM-MSCs combined with BM-MNCs show greater promise in long-term complication prevention. While BM-MSCs have a well-established track record in clinical applications and possess remarkable multipotent differentiation capabilities, their invasive harvesting process remains a limitation [48]. In contrast, UC-MSCs and WJ-MSCs have gained attention as promising alternatives due to their non-invasive accessibility, low immunogenicity, and robust proliferative capacity, making them highly suitable for diabetes treatment [49]. Moving forward, further research is essential to refine the isolation, cultivation, and application strategies of these MSCs, ensuring their full therapeutic potential is realized in the management of diabetes.

## Application of MSC-based therapy in complications of diabetes

Recent preclinical and clinical studies highlight the therapeutic potential of MSCs in mitigating diabetes-related complications through mechanisms such as paracrine signaling, immunomodulation, and extracellular vesicle (EV)-mediated effects. Below, we summarize key advancements in MSC-based therapies for major diabetic complications, supported by clinical trials from the past decade (Table 4) [50–54].

**Table 4:** MSC-based therapy for diabetes complications in clinical trials

Complication	Subjects (n)	Stem cell type	Follow-up	Baseline characteristics	Key outcomes	Trial registration	References
DNp	16	BM-MSCs	18 months	Albuminuria (UACR ≥ 88 mg/g) Impaired kidney function (eGFR 25–55 ml/min/1.73 m^2^)	Vs. Placebo: Safe and well-tolerated Comparable mGFR decline (*p* = 0.467) Slower eGFR decline by CKD-EPI/MDRD (*p* = 0.034)	NCT02585622	[50]
Diabetic foot ulcers	28	BM-MSCs	once a week until wound closure	Grade 1 or 2 Ulcer area: 0.5–5.5 cm^2^	Vs. PolyMem Safe Improved wound healing and ulcer-free survival (*p* < 0.05)	NCT124174455522	[51]
	14	UC-MSC	3 years	Wagner grade ≥ 3 Ulcer Size ≥ 3 cm^2^	Safe and effective 95% ulcer reduction within 1.5 months Chronic limb ischemia symptoms improvement: reduced Wagner (*p* = 0.001), Rutherford (*p* = 0.003), and VAS scores from baseline (*p* < 0.001)	ChiCTR2200055885	[52]
	23	Skin-derived ABCB5 + MSCs	12 months	Wagner grade 1–2 Median area: 1.9 cm^2^	Safe Median ulcer area reduction from baseline at 12 weeks: 59% (full analysis set), 64% (per-protocol set), and 67% (responder subgroup)	NCT03267784	[53]
	2	BM-MSCs	7 months	Ulcer areas 50 mm^2^ and 130 mm^2^	Acceptable safety profile Transient increased ulcer exudation No confirmed treatment-related serious adverse events	EudraCT number 2015–005580-16	[54]

### MSC-based therapy for DW

DW is a common complication of diabetes, accounting for approximately 20–25% of the reasons for hospitalization among diabetic patients [55]. The core pathological mechanisms include: (1) microcirculation disorders and neuropathy induced by a high-glucose environment; (2) a chronic inflammatory state accompanied by persistently elevated pro-inflammatory factors (IL-6, TNF-α), and (3) impaired angiogenesis [56, 57]. Current treatment methods, such as debridement and negative pressure wound therapy, have limited efficacy in addressing the underlying pathologies, and the amputation rate remains as high as 15–20% [56].

Recent studies demonstrate that MSCs promote DW through synergistic multimodal mechanisms. Regarding immunomodulation, C–C motif chemokine receptor 2-modified MSCs exhibit superior performance relative to monocyte infiltration, macrophage polarization, and Treg accumulation (*p* < 0.01) [58].

For angiogenesis, The MSCs/FG (a composite of MSCs in a fibrin scaffold) therapy boosted vascular density (*p* < 0.01) and fostered functional microvessel (CD34 + α-SMA +) formation (*p* < 0.01) by activating the Akt/mTOR-VEGF pathway [59].

In tissue regeneration, AD-MSCs display unique regenerative attributes: when combined with platelet-rich plasma, they increase collagen area and epidermal thickness via Notch pathway while markedly recruiting epidermal stem cells [60]. Remarkably, even AD-MSCs derived from diabetic individuals maintain normal healing capacity, significantly elevating type I/III collagen and CD31 expression (all *p* < 0.05) [61].

Emerging research highlights the critical role of MSC-derived exosomes: they simultaneously restore transient receptor potential cation channel subfamily C member 6 and mitochondrial function by delivering SP2/USP9 to correct calcium dyshomeostasis, while activating Notch signaling to promote CD31 + EMCN + vascular regeneration [56, 62].

The transformative potential of this approach has been substantiated through multiple clinical trials. Studies indicate that MSCs, derived from various sources and delivered through different administration routes, consistently demonstrate favorable therapeutic outcomes in diabetic foot ulcers. Percutaneous injection of BM-MSCs significantly enhanced wound healing rates compared to PolyMem treatment (*p* < 0.01) (NCT124174455522) [51]. Intravenous infusion of UC-MSCs facilitated rapid ulcer closure and provided sustained alleviation of ischemic symptoms in severe cases, with notable improvements in chronic limb ischemia as reflected by reductions in Wagner (*p* = 0.001), Rutherford (*p* = 0.003), and VAS scores (*p* < 0.001) from baseline (ChiCTR2200055885) [53]. Additionally, topical administration of skin-derived ABCB5 + stem cells leads to a substantial median reduction in refractory ulcer area from baseline, ranging from 59 to 67% over 12 weeks (NCT03267784) [52]. Regarding safety, available evidence suggests a low incidence of treatment-related adverse events, which are predominantly mild and localized in nature [54].

Future research should focus on larger-scale, multicenter randomized controlled trials to further validate the efficacy and safety of MSCs, as well as explore their combined use with other treatment modalities (such as debridement and negative pressure wound therapy) to further enhance therapeutic outcomes, offering new hope for improving patients' quality of life and reducing amputation rates.

### MSC-based therapy for DNp

DNp, as one of the most devastating microvascular complications of diabetes, poses a significant clinical challenge worldwide. Epidemiological data indicate that DNp accounts for 40–50% of end-stage renal disease (ESRD) cases in Western countries, highlighting its substantial public health burden [63]. Characteristic pathological features include glomerular basement membrane thickening, mesangial matrix expansion, and progressive glomerulosclerosis, clinically manifested as proteinuria, hypertension, and progressive renal function decline [64, 65]. Although current standard therapies (including strict glycemic control, blood pressure management, and renin-angiotensin system inhibitors) can partially slow disease progression, they fail to reverse established renal structural damage [66]. Alarmingly, approximately 30–40% of patients inevitably progress to ESRD, requiring renal replacement therapy. This critical situation underscores the urgent need for novel renal-protective therapeutic approaches.

In recent years, MSC-based therapy has attracted considerable attention due to its unique therapeutic properties. Extensive preclinical studies have elucidated the molecular mechanisms through which MSCs exert renal protection via multiple targets and pathways: (1) Immunomodulatory effects: Recent studies demonstrate that MSC-derived miR-146a-5p significantly reduces renal levels of pro-inflammatory cytokines (IL-6, TNF-α, and IL-1β; *p* < 0.01) by specifically inhibiting the TNF receptor associated factor 6/STAT1 signaling pathway [67]; (2) Anti-fibrotic effects: MSCs upregulate solute carrier family 3 member 2 expression to effectively suppress ferroptosis. Concurrently, they inhibit the MAPK signaling pathway (including ERK, JNK, and p38 phosphorylation), coordinately downregulating key fibrotic markers (transforming growth factor-β (TGF-β), α-SMA, and collagen I, *p* < 0.05), thereby significantly ameliorating renal fibrosis [68]; (3) Antioxidant capacity: Researchers led by Nie found that UCMSCs activate the PI3K-Akt pathway to promote nuclear translocation of transcription factor Nrf2, subsequently upregulating antioxidant enzymes (SOD2, HO-1, and NQO1; *p* < 0.05), effectively mitigating oxidative damage in DNp [69]; (4) Mitochondrial function restoration: Emerging mechanistic studies reveal that MSCs repair mitochondrial dysfunction in injured renal tubular epithelial cells through an arginase-1-dependent pathway, providing novel insights into MSC-mediated renal protection [70].

In clinical translation, the NEPHSTROM trial (a randomized, double-blind, placebo-controlled phase 1b/2a study) has yielded promising results (NCT02585622). This study evaluated next-generation, CD362 antibody-selected allogeneic MSCs in 16 type 2 diabetic patients with progressive DNp. Safety analysis showed no severe infusion-related adverse events in the treatment group. Compared with placebo, MSC-treated patients exhibited significantly slower annual estimated glomerular filtration rate decline (*p* < 0.05), providing preliminary evidence of renal protective potential [50].

### MSC-based therapy for DR

DR is a sight-threatening complication of diabetes characterized by blood-retinal barrier (BRB) breakdown, pericyte loss, and progressive retinal ischemia, ultimately leading to pathological angiogenesis. The core pathogenesis of DR involves synergistic damage to the neurovascular unit [71]. While traditionally classified as a microvascular disorder, emerging evidence reveals distinct neurovascular sequential injury characteristics: retinal neurodegeneration precedes vascular pathology, manifesting as progressive atrophy in the nerve fiber layer (annual loss rate 0.25 μm) and ganglion cell layer (annual loss rate 0.29 μm) [72]. This paradigm shift redefines DR classification frameworks, suggesting neuroprotective interventions may offer earlier therapeutic windows.

Current anti-VEGF therapies, though effective in delaying end-stage vascular complications, face critical limitations: primary non-response occurs in ~ 30% of patients, with no capacity to reverse established neurodegenerative changes [73]. These constraints underscore the urgent need for novel strategies targeting upstream pathological processes.

MSCs exert multidimensional therapeutic effects via paracrine regulation, with mechanisms systematically categorized as: (1) BRB restoration: AD-MSCs differentiate into pericyte-like cells, enhancing tight junction proteins (zona occludens-1/vascular endothelial cadherin) while suppressing inflammatory mediators (TNF-α/IL-1β/matrix metalloproteinase-9), significantly reducing vascular permeability (*p* < 0.01) [74]; (2) Neuroprotective action: Intravitreal MSC administration secretes brain-derived neurotrophic factor (BDNF) and glial cell line-derived neurotrophic factor (GDNF) neurotrophic factors, markedly decreasing retinal ganglion cell (RGC) apoptosis (*p* < 0.05) [75].

To address low in vivo MSC survival rates, MSC-derived EVs have emerged as promising delivery vehicles. BM-MSC EVs transport miR-5068/miR-10228 to inhibit HIF-1α/enhancer of zeste homologue 2 (EZH2) signaling, reversing peroxisome proliferator-activated receptor-γ coactivator-1α (PGC-1α) epigenetic silencing via histone H3 lysine 27 trimethylation modification, thereby ameliorating hyperglycemia-induced metabolic-vascular damage [76]. UC-MSC EVs containing miR-17-3p suppress STAT1 pathways [77]. Ebrahim et al. demonstrated EV-mediated Wnt/β-catenin pathway blockade attenuates oxidative stress, inflammation, and pathological angiogenesis [78].

Critical challenges remain in clinical translation: Administration routes require optimization between intravitreal injection specificity and systemic effects of intravenous delivery; EV engineering needs improved miRNA loading efficiency (> 80%); Safety protocols must establish TGF-β1 monitoring thresholds (< 50 pg/mL) to quantify long-term fibrotic risks. Resolution of these issues will directly determine the clinical viability of these innovative therapeutic approaches.

### MSC-based therapy for DNe

DNe, the most prevalent chronic complication of diabetes, is characterized by progressive distal-to-proximal degeneration of peripheral nerves [79]. Clinical manifestations encompass sensory abnormalities, neuropathic pain, and foot ulcers, with an incidence reaching 50%, significantly elevating mortality risk and impairing quality of life [80]. The pathogenesis involves multifaceted mechanisms where chronic hyperglycemia induces neural damage through oxidative stress, advanced glycation end-products accumulation, and other pathways, with distal symmetric polyneuropathy being the predominant subtype [81].

Current diagnostic approaches follow ADA guidelines incorporating composite assessments including 10 g monofilament testing, vibratory perception examination, and nerve conduction velocity measurements [82]. Notably, approximately 30% of patients exhibit disease progression despite optimal glycemic control (HbA1c < 7%), underscoring the limitations of existing therapies and highlighting the urgent need for novel interventions targeting key pathological mechanisms [83].

Emerging evidence suggests MSCs exert therapeutic effects through paracrine modulation and activation of critical signaling cascades. By secreting bioactive factors (VEGF, basic fibroblast growth factor (bFGF), nerve growth factor) and EVs, MSCs remodel the neural microenvironment, achieving anti-inflammatory, pro-angiogenic, and anti-apoptotic outcomes [84]. For instance, conditioned medium from AD-MSCs mitigates neuroinflammation by upregulating neuroprotective mediators GDNF, consequently improving sensory dysfunction and cutaneous reinnervation in murine models [85].

Notably, tissue-specific MSC populations engage distinct molecular pathways. BM-MSCs predominantly activate GSK-3β/β-catenin signaling to enhance nerve conduction velocity and vascular regeneration [86], whereas placental MSCs (PMSCs) utilize the dishevelled1/β-catenin axis to promote remyelination [87]. This pathway selectivity likely stems from microenvironmental responsiveness, though precise regulatory networks require further elucidation.

Preclinical studies demonstrate treatment efficacy correlates strongly with targeted delivery efficiency and microenvironmental compatibility. Intramuscular BM-MSC administration markedly increases sciatic nerve blood flow via localized VEGF/bFGF upregulation [88]. However, intravenous infusion shows reduced effectiveness due to limited homing capacity, emphasizing route optimization as a critical translational consideration.

The identified Wnt pathway involvement in PMSC therapy offers novel mechanistic insights. While β-catenin nuclear translocation facilitates Schwann cell regeneration, Wnt signaling's dual roles in inflammatory modulation and metabolic regulation necessitate validation using gene-edited models. These complexities suggest MSC-mediated recovery likely involves coordinated multi-pathway interactions rather than isolated molecular events.

Despite therapeutic promise, clinical translation faces three major challenges: (1) Existing evidence demonstrates predominantly short-term symptomatic amelioration, with the long-term neurorestorative capacity requiring further validation; (2) Secretory profile variations across MSC sources may impact reproducibility; (3) Current mechanistic studies predominantly rely on inhibitors/overexpression approaches lacking in vivo dynamic monitoring.

Future investigations should integrate single-cell sequencing with live imaging to decipher three-dimensional MSC-host interactions within neural/vascular/immune niches. Concurrently, developing engineered MSCs (e.g., neurotrophic factor-overexpressing variants) may overcome current efficacy limitations.

### MSC-based therapy for DCM

Rubler et al. first identified DCM in an autopsy study in 1972 [89]. DCM is a chronic diabetic complication that develops as a result of long-term uncontrolled high blood sugar [90]. DCM is exclusively linked to diabetes as its singular risk factor, showing no correlation with coronary artery disease, hypertension, or severe valvular disease [91]. Hyperglycemia initiates cardiac structural remodeling, cardiomyocyte apoptosis, and activation of systemic and tissue RAAS through the involvement of various signaling pathways. These pathways include protein kinase C, MAPK, NF-κB, SGLT 2, O-GlcNAc, and CREM, which collectively contribute to the development of DCM [92]. The complex pathophysiological mechanisms that contribute to the progression of DCM include a range of factors such as systemic metabolic disorders, inappropriate activation of the renin–angiotensin–aldosterone system, subcellular component abnormalities, oxidative stress, inflammation and dysfunctional immune modulation [93]. DCM is characterized by initial diastolic dysfunction, which progresses to systolic dysfunction over time [94]. DCM eventually leads to heart failure (HF), marked by cardiac hypertrophy, interstitial fibrosis, increased capillary basement membrane thickness, capillary microangioma, and decreased capillary density [92]. During its early stages, DCM is classified as a type of Stage B HF, defined as structural heart disease without past or present overt HF [89].

The Aldose Reductase Inhibition for Stabilization of Exercise Capacity in HF Trial, spearheaded by James L. Januzzi and colleagues, involved a cohort of 691 individuals with T2D. This pivotal study delved into the baseline characteristics of a group of patients with T2D, Stage B HF and found that many patients showed elevated biomarkers and abnormal echocardiogram, which could help early identification and intervention with DCM in clinical practice [89]. Although glycemic control has traditionally been a cornerstone of CDM therapy, multicenter randomized clinical studies have demonstrated that intensive glycemic control does not lead to a reduction in HF-related hospitalization and mortality among patients with diabetes [92]. A rapid surge in both preclinical and clinical investigations focused on DCM in recent years, but an evidence-based, universally accepted treatment is still not on the horizon.

**Fig. 4:**
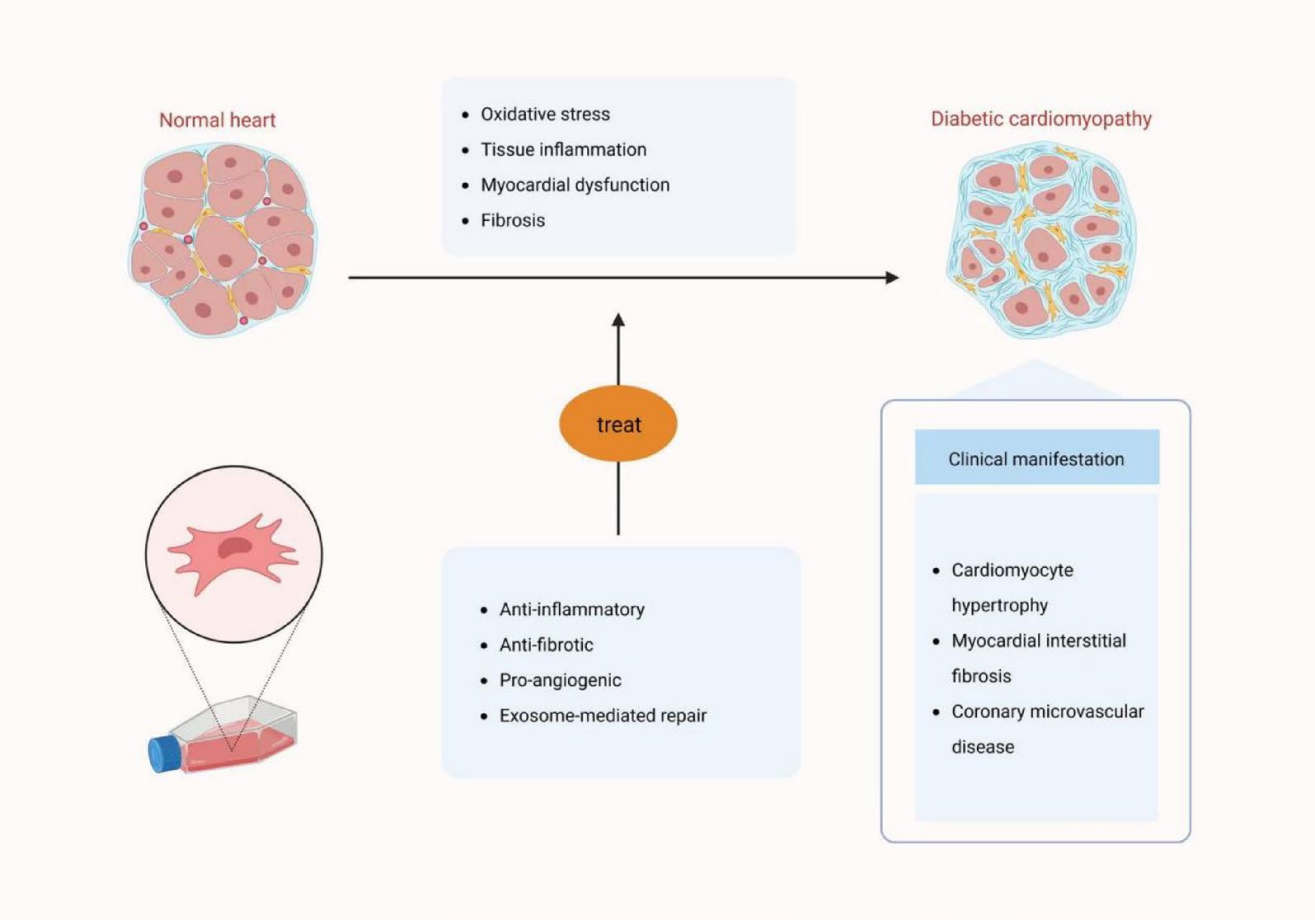
MSCs ameliorate DCM via multi-target mechanisms. This figure depicts the interplay of RAAS dysregulation, oxidative stress, and MSC-mediated anti-inflammatory and anti-fibrotic effects in DCM. Created in BioRender. Tu, L. (2025) https://BioRender.com/2eusplt

As shown in Fig. 4, MSCs hold substantial potential for the treatment of DCM. Cumulative preclinical studies have demonstrated that MSCs can improve cardiac dysfunction and post-infarction vascular remodeling [95]. A study has shown that MSC-Exos therapy can effectively reverse cardiac damage caused by inflammation, necroptosis activation, and increased expression of TAK 1, pJNK, NF-κB. Compared to diabetic hyperlipidemic mice, MSC-Exos treated mice displayed decreased expression of markers associated with necrotic apoptosis [96]. In a separate study, MSCs have been shown to promote the polarization of M2 macrophages, suppress the proinflammatory response, and increase anti-inflammatory cytokines to reduce inflammation in the heart affected by DCM. The subsequent in vitro experiments conducted by the authors further validated this discovery and demonstrated that MSCs facilitate M2 macrophage polarization via the COX-2-PGE2 pathway [97]. Moreover, the researchers have identified that MSCs exert regulatory control over CDM via a competing endogenous RNA network involving circNOTCH 1, miR-495-3p, and NOTCH 1. Exosomes derived from ginsenoside RG1 (RG1)-induced MSCs promote the transfer of circNOTCH1 into macrophages, activating NOTCH signaling pathway and subsequently driving macrophage polarization towards the M2 phenotype [90].

In addition, MSCs exert anti-fibrotic influences by diminishing cardiac fibroblast activity and collagen deposition. The co-culture experiments of myocardial fibroblasts and MSCs in vitro demonstrated that the anti-fibrotic effect of MSCs was associated with the secretion of prostaglandin E2 [98]. Nonetheless, the efficacy of MSCs in mitigating myocardial fibrosis warrants further enhancement and refinement. Diabetes leads to compromised bioactivity and repair capacity of BM-MSCs [99]. Therefore, Ke Meng et al. utilized adiponectin (APN) plasmid or APN small interfering RNA to modify BM-MSCs, and investigated its mechanism of action on myocardial fibrosis in diabetic rats both in vitro and in vivo. Their results showed that the APN modified BM-MSCs alleviated myocardial myofibrillar disorder, suppressed proliferation and transformation of myocardial fibroblasts, and inhibited myocyte/fibroblast interactions via the TGF-β1/Smad signaling pathway [100]. Consequently, the anti-diabetic features of MSCs, along with their cardioprotective properties, render their application a promising therapeutic strategy for DCM.

### Comparative discussion

MSC-based therapies, beyond their common mechanisms like immunomodulation, exhibit unique therapeutic priorities across different complications: diabetic wounds focus on tissue regeneration, diabetic nephropathy emphasizes ferroptosis inhibition and metabolic reprogramming, diabetic retinopathy highlights blood-retinal barrier repair and neuroprotection, diabetic neuropathy primarily targets microenvironment remodeling and remyelination, while diabetic cardiomyopathy addresses structural damage repair. The uneven progress in their clinical translation profoundly reflects the close correlation between efficacy and the pathological characteristics of target tissues, as well as the clinical evaluation system (as shown in Table 5). A striking contrast is evident between DW and DCM. This disparity primarily stems from three fundamental reasons: First, in terms of tissue accessibility and microenvironment, the skin, as a superficial organ, allows direct delivery via local injection or scaffolds, ensuring high local concentration of therapeutic factors in a microenvironment conducive to repair. In contrast, the heart, as a deep and complex organ, relies on systemic intravenous infusion, resulting in extremely low homing efficiency. Coupled with a hostile pathological microenvironment featuring myocardial fibrosis, chronic inflammation, and metabolic dysregulation, the survival and function of MSCs are severely compromised. Second, regarding the match with pathological mechanisms, the core functions of MSCs—promoting angiogenesis and tissue remodeling—perfectly align with the central issue of "impaired repair" in DW. However, for DCM, whose core pathology is "progressive structural damage," the anti-inflammatory and anti-fibrotic effects of MSCs can modulate the disease process but are insufficient to reverse established cardiomyocyte loss and extensive fibrosis. Finally, in terms of clinical translation feasibility, the efficacy of DW treatments can be rapidly assessed within weeks or months using straightforward indicators such as wound closure rates, greatly accelerating clinical validation. In contrast, the evaluation of DCM requires reliance on long-term and complex endpoint events such as cardiac functional decline and heart failure hospitalization rates, leading to longer, more costly, and higher-risk clinical trials.

**Table 5:** Summary of MSC therapeutic mechanisms and clinical advances across diabetic complications

Complication	Mechanisms of MSC action	Key signaling pathways	Key molecules/ mediators	Clinical trial evidence
DW	Tissue Regeneration	Akt/mTOR, Notch	PD-L1, IDO, IL-10, VEGF-A, HGF, SP2, USP9	Multiple clinical trials using various routes (IV, intradermal) report significantly improved wound closure rates, skin regeneration, and ulcer-free survival with good safety and long-term (e.g., 3-year) efficacy
DNp	Cell Death Intervention & Metabolic Reprogramming	TRAF6/STAT1, MAPK (ERK, JNK, p38), PI3K-Akt/Nrf2	IL-10, TSG-6, miR-146a-5p,	The NEPHSTROM trial (Phase Ib/IIa, randomized, double-blind, placebo-controlled) showed that CD362-selected allogeneic MSCs significantly slowed the annual decline of eGFR with a good safety profile
DR	Neuro-Vascular Unit Protection	HIF-1α/EZH2, STAT1, Wnt/β-catenin	BDNF, GDNF, miR-5068, miR-10228, miR-17-3p	Primarily preclinical. Challenges include optimizing delivery route (intravitreal vs. intravenous), improving EV miRNA loading efficiency, and establishing long-term safety monitoring (e.g., TGF-β1 thresholds)
DNe	Microenvironment Remodeling & Re-myelination	GSK-3β/β-catenin, Dishevelled1/β-catenin	VEGF, bFGF, NGF, IL-10, GDNF	Limited clinical evidence; mostly preclinical. Challenges include demonstrating long-term efficacy, heterogeneity of MSC secretory profiles, and optimizing targeted delivery (e.g., intramuscular > intravenous)
DCM	Counteracting Structural Myocardial Damage	COX-2-PGE2, NOTCH, TGF-β1/Smad	PGE2, IL-10,TGF-β1	Evidence is primarily preclinical. MSC-derived exosomes can reverse cardiac inflammation and necroptosis. Engineering (e.g., adiponectin modification) enhances anti-fibrotic effects

Looking ahead, future research in this field should focus on optimizing targeted delivery strategies, developing engineered MSCs to overcome microenvironmental limitations, and establishing standardized treatment protocols and evaluation systems. These efforts will drive MSC-based therapies toward more precise and efficient clinical applications, bringing new hope to more patients with diabetes.

## The challenges of MSC-based therapy for diabetes treatment

MSC-based therapy holds promise for diabetes treatment, yet faces significant translational challenges. The promising efficacy observed in preclinical models has not been fully replicated in human trials. This discrepancy arises from several factors: (1) Preclinical limitations: rodent models using STZ/high-fat diets fail to replicate human pathophysiology and comorbidities; (2) Clinical constraints: patient heterogeneity, safety-limited dosing, and shorter follow-ups versus animal studies.

Based on current clinical evidence, the impact of individual patient factors on the efficacy of MSC therapy for diabetes is not uniform. Age, disease duration, body mass index (BMI), and comorbidities are key determining factors: younger patients with a short disease duration (e.g., < 10 years) and non-obese status (BMI < 23 kg/m^2^) show the best response, due to their relatively favorable metabolic and inflammatory environment [101]. In contrast, advanced age accompanied by "inflammaging", long-term hyperglycemia, and obesity-related chronic inflammation can impair MSC function and diminish therapeutic efficacy [102, 103]. The influence of sex appears relatively limited and inconsistent, with short-term (9 weeks) observed differences often not persisting in long-term (48 weeks) follow-up [43]. In summary, treatment efficacy is highly dependent on the patient's internal environment. Optimizing host metabolic status and implementing precise patient stratification are core strategies for improving therapeutic success, while future efforts should focus on developing enhanced treatment regimens for high-risk groups.

These challenges are compounded by MSC heterogeneity. This variability arises from donor-specific differences, tissue source variations (e.g., adipose vs. bone marrow), and dynamic changes during in vitro expansion, ultimately leading to significant batch-to-batch disparities in proliferation capacity, differentiation potential, and critically, secretory profiles [104]. Clinical evidence underscores this heterogeneity. In the Phase III PROCHYMAL trial (involving multi-donor BM-MSCs) for acute graft-versus-host disease, the treatment group failed to meet the primary endpoint. Post-hoc analysis identified variations in IDO activity among donors as a key factor driving inconsistent outcomes, highlighting the direct impact of functional donor heterogeneity on clinical reproducibility [105]. Differences in tissue sources may also affect clinical treatment outcomes. One randomized controlled trial reported that 82% of patients receiving BM-MSCs achieved a ≥ 50% reduction in insulin requirement, with nearly 67% maintaining long-term benefits. In contrast, clinical data on AD-MSCs remain limited. The sole reported Chinese clinical trial indicated superior glycemic control compared to conventional therapy, yet long-term efficacy data await further validation [106, 107].

To address these challenges, single-cell RNA sequencing is being employed to systematically deconstruct and ultimately reduce MSC product variability. This technology resolves conventionally homogeneous MSC populations into distinct subpopulations with unique gene expression signatures, such as stem-like, functional, and proliferative clusters [108]. Identifying and isolating specific therapeutically potent subpopulations (e.g., immunomodulatory subsets highly expressing TGF-β) could potentially replace conventional unsorted MSCs, yielding more functionally uniform and predictably efficacious products. However, this approach faces considerable hurdles: the limited expansion capacity of sorted cells may fall short of clinical dosing requirements; the phenotypic stability of therapeutic subsets within the in vivo microenvironment remains uncertain; and the high costs and complex processes pose significant challenges for scaling up production under current Good Manufacturing Practice standards.

The diabetic milieu presents additional formidable barriers. Chronic hyperglycemia impairs MSC survival by disrupting mitochondrial dynamics and modulating key regulatory proteins in the PI3K/mTOR pathway, ultimately leading to enhanced apoptotic cell death [109–111].

To overcome these limitations, innovative engineering strategies are being developed: (1) Genetic enhancement: This strategy aims to endow MSCs with endogenous capabilities to resist endoplasmic reticulum stress, oxidative stress, and apoptosis via genetic engineering. For instance, Exendin-4-modified MSCs activate the GLP-1R/AMPK pathway through an autocrine mechanism, significantly enhancing their survival under hyperglycemic stress, while concurrently inhibiting pancreatic β-cell senescence and apoptosis via endocrine actions [112]. Clinical translation, however, faces safety concerns: retroviral or lentiviral vectors carry risks of insertional mutagenesis and proto-oncogene activation. Although non-viral vectors (e.g., transposons) and inducible expression systems are under development, their transfection efficiency and long-term expression stability require optimization. Furthermore, tumorigenicity assessment of genetically modified cells necessitates extensive long-term follow-up studies, substantially increasing the cost and timeline of clinical development. (2) Preconditioning strategies: Hypoxic preconditioning stabilizes the HIF-1α transcription factor, initiating a cascade of adaptive cellular responses, including upregulation of pro-survival factors (e.g., B-cell lymphoma 2), enhanced glycolytic metabolism, and reprogramming of the secretome to enrich potent pro-angiogenic and anti-inflammatory factors. Studies demonstrate that preconditioned exosomes exhibit remarkable efficacy in diabetic wound models, accelerating healing by 30–40% (*p* < 0.001) while substantially reducing oxidative stress (50% increase in ROS clearance, *p* < 0.001) and inflammation (60–70% reduction in cytokine levels, *p* < 0.001) [113]. 3D culture, by reconstituting cell–cell and cell–matrix interactions, mimics the native stem cell niche, effectively preserving MSC stemness and paracrine function. In a diabetic nephropathy model, 3D MSC spheroids significantly enhanced the secretion of angiogenic factors like VEGF and HGF compared to traditional 2D-cultured cells, leading to approximately 30% improvement in renal function indicators [114]. While hypoxic preconditioning is readily scalable in bioreactors, 3D culture—particularly for large-scale organoids—faces challenges in process monitoring and product homogeneity. A critical bottleneck for clinical translation is the establishment of robust "potency biomarkers" (e.g., specific miRNA or protein secretion profiles) directly correlated with efficacy, aiming to replace current quality control standards primarily based on cell count and viability, thereby ensuring batch-to-batch consistency and therapeutic potential of preconditioned cells. (3) Biomaterial encapsulation: This technology enhances MSC therapy through a dual mechanism: the material acts as a physical barrier against host immune attacks, while fine-tuning material stiffness, degradation rate, and incorporated biological signals can precisely direct the survival, paracrine activity, and differentiation of encapsulated cells [115]. However, clinical advancement is constrained by material biocompatibility and long-term safety concerns: implants may elicit fibrotic encapsulation, hindering nutrient/waste exchange and molecular diffusion; non-degradable materials might require surgical removal; and the safety profiles of degradation products from biodegradable materials require comprehensive evaluation. Furthermore, real-time, non-destructive monitoring of the viability and function of encapsulated cells remains a significant technical hurdle.

Collectively, these advances are paving the way for more predictable and durable clinical outcomes.

## Conclusion and outlook

The therapeutic potential of MSCs in diabetes and its complications is advancing into clinical translation, demonstrating benefits through modulation of immune dysregulation, β-cell function, and metabolic homeostasis. While clinical studies have established the short-term safety of MSC-based interventions and suggested potential for β-cell functional improvement, the durability of these effects and the standardization of treatment protocols remain significant challenges.

Notably, the therapeutic efficacy observed in human trials has generally been more modest and variable compared to the robust effects consistently reported in preclinical models. This translational gap underscores the limitations of current animal models and the complexity of human diabetes pathophysiology. To address these limitations, future research must integrate multidimensional innovations: Mechanistically, leveraging single-cell sequencing to identify high-potency subpopulations and engineering genetically modified cells co-expressing GLP-1/FGF21 or ROS-responsive microencapsulation systems could enable precise microenvironmental regulation.

Translationally, overcoming the barriers to clinical adoption requires a concerted focus on three fronts. Manufacturing-wise, the development of closed, automated bioreactor systems and universally accepted potency assays is essential to ensure batch-to-batch consistency and meet Good Manufacturing Practice standards, thereby addressing a key regulatory hurdle. Clinically, designing robust Phase III trials with standardized efficacy endpoints—such as C-peptide response combined with insulin dosage reduction—is crucial for generating compelling data for both regulators and payers. Furthermore, establishing the therapy's cost-effectiveness through real-world evidence and health economic studies will be pivotal for securing reimbursement. Finally, the exploration of "off-the-shelf" products, particularly lyophilized MSCs, represents a promising strategy to dramatically improve logistical feasibility and reduce costs, accelerating widespread accessibility.

In conclusion, while MSC-based therapies represent a significant and innovative avenue in diabetes research, their current status is that of a promising yet unproven therapeutic modality. The path to clinical integration hinges not only on scientific and technological breakthroughs but also on transparent reporting of clinical outcomes, including null results and limitations. A measured, evidence-based perspective is crucial for responsibly advancing this field toward its goal of modifying the natural history of diabetes.

## Data Availability

No data was used for the research described in the article.

## Abbreviations

MSCs: Mesenchymal stromal cells
UC-MSCs: Umbilical cord-derived MSCs
BM-MSCs: Bone marrow-derived MSCs
WJ-MSCs: Wharton’s jelly MSCs
DW: Diabetic wounds
DNp: Diabetic nephropathy
DR: Diabetic retinopathy
DNe: Diabetic neuropathy
DCM: Diabetic cardiomyopathy
SGLT2: Sodium-glucose co-transporter 2
miR-126: MicroRNA-126
T1D: Type 1 diabetes
T2D: Type 2 diabetes
GDM: Gestational diabetes mellitus
PD-1: Cell death protein 1
PD-L1: Programmed death-legend 1
IDO: Indoleamine 2,3-dioxygenase
AD-MSCs: Adipose-derived MSCs
TNF-α: Tumor necrosis factor-α
IL: Interleukin
PGE2: Prostaglandin E2
TSG-6: Tumor necrosis factor-inducible gene 6 protein
STAT1: Signal transducer and activator of transcription 1
CCL22: C–C motif chemokine ligand 22
VEGF: Vascular endothelial growth factor
HGF: Hepatocyte growth factor
DFX-CM: Deferoxamine-pretreated MSCs
IPCs: Insulin-producing cells
FGF21: Fibroblast growth factor 21
GLP-1: Glucagon-like peptide-1
AEs: Adverse events
aBM-MNCs: Autologous bone marrow mononuclear cells
AUCC-Pep: C-peptide area under the curve
HbA1c: Glycosylated hemoglobin
TIR: Time-in-range
EVs: Extracellular vesicles
HIF-1α: Hypoxia-inducible factor-1α
ESRD: End-stage renal disease
TGF-β: Transforming growth factor-β
BRB: Blood-retinal barrier
BDNF: Brain-derived neurotrophic factor
GDNF: Glial cell line-derived neurotrophic factor
RGC: Retinal ganglion cell
EZH2: Enhancer of zeste homologue 2
PGC-1α: Peroxisome proliferator-activated receptor-γ coactivator-1α
bFGF: Basic fibroblast growth factor
PMSCs: Placental MSCs
HF: Heart failure
RG1: Ginsenoside RG1
APN: Adiponectin
BMI: Body mass index
